# Influence of Synthesis Conditions on Microstructure and NO_2_ Sensing Properties of WO_3_ Porous Films Synthesized by Non-Hydrolytic Sol–Gel Method

**DOI:** 10.3390/nano9010008

**Published:** 2018-12-21

**Authors:** Sikai Zhao, Yanbai Shen, Pengfei Zhou, Guodong Li, Cong Han, Dezhou Wei, Xiangxi Zhong, Yunhai Zhang, Yuxin Ao

**Affiliations:** 1 School of Resources and Civil Engineering, Northeastern University, Shenyang 110819, China; sikaizhao@gmail.com (S.Z.); pengfei.zhou1710387@gmail.com (P.Z.); liguodong2015@163.com (G.L.); cong.han1@gmail.com (C.H.); dzwei@mail.neu.edu.cn (D.W.); zhongxx007@gmail.com (X.Z.); 2 State Key Laboratory of Mineral Processing, Beijing, 102628, China; bradleyzhang@163.com; 3 School of Mechanical and Electrical Engineering, Shenyang Aerospace University, Shenyang 110136, China; aoyuxin@gmail.com

**Keywords:** WO_3_, porous films, non-hydrolytic sol–gel, NO_2_, gas sensing

## Abstract

Nanostructured tungsten trioxide porous films were prepared by a non-hydrolytic sol–gel method following the inorganic route in which ethanol and PEG were used as the oxygen-donor and structure-directing reagent, respectively. The effects of aging time of the precursor solution, PEG content, and calcination temperature on the structure, morphology, and NO_2_ sensing properties of WO_3_ films were systematically investigated by using the techniques of X-ray diffraction, field emission scanning electron microscopy, transmission electron microscopy, and gas sensing measurements. The results demonstrated that a series of WO_3_ films with different microstructures could be obtained by manipulating the synthesis parameters. Furthermore, a suitable synthesis condition of WO_3_ films for NO_2_ sensing application was determined.

## 1. Introduction

Tungsten trioxide (WO_3_), as an important *n*-type semiconductor metal oxide with a wide band gap of 2.6~3.0 eV [1], is showing promising applications in a wide number of novel products, including photocatalytic [2], electrochromic devices [3], dye-sensitized solar cells [4], optical devices [5], field-emission displays [6], and gas sensors [7,8,9]. In particular, WO_3_ is widely considered to be one of the most promising alternative candidate materials for the next-generation gas sensing devices [10,11].

In 1967, Shaver observed the change in conductivity of WO_3_ films upon exposure to H_2_ atmosphere [12]. From then on, the application of WO_3_ films in gas sensing has been attracting great attention. Especially in recent years, nanostructured WO_3_ films have been the subject of intense research for high-performance NO_2_ sensing due to their excellent NO_2_ selectivity. Shen et al. prepared WO_3_ thin films with different effective surface areas by using reactive magnetron sputtering and investigated their relationship between the effective surface areas and NO_2_ sensing performance [13]. Lozzi et al. investigated the NO_2_ sensing properties of WO_3_ thin films that thermally evaporated on Si_3_N_4_/Si substrates [14]. Ponzoni et al. reported a modified evaporation method to obtain WO_3_ thin films with high surface roughness for gas sensing applications [15]. Penza et al. investigated the NO_x_ sensing properties of WO_3_ thin films fabricated on glass substrates by reactive RF sputtering [16]. Although these efforts have been made to give a deep insight into the NO_2_ sensing performance of WO_3_ films, it is evident that these preparation methods are mainly centered on sputtering and evaporation, in which expensive equipment and high temperature are required. In addition, the films synthesized by these methods are relatively dense and the yield is low. Therefore, a simple and cost-effective preparation route is urgently demanded for the further development of WO_3_ films in gas sensing application.

Sol−gel chemistry is one of the most effective and suitable routes for preparing nanocomposite films from the liquid phase due to their intrinsic advantages, such as low preparation temperature, low cost of fabrication facilities, uniform deposit with desired shapes, possibility of a precise stoichiometry and thickness control [17,18,19]. The conventional sol–gel process based on the formation of oxo bridge includes two major steps: (1) hydrolysis of sol–gel precursor; (2) polycondensation of the hydrolyzed products and the other sol–gel active groups presenting in the system [20]. However, for the conventional sol–gel process, it is not straightforward to control the composition, morphology, and structure of the final products simultaneously, especially in the case of a mixed oxide system where the reaction rates of the different precursors have to be matched in order to get homogeneous mixed oxide gel [21]. In order to overcome these shortcomings, the non-hydrolytic sol–gel (NHSG) chemistry, in which the oxo bridges originate from oxygen-donors rather than water, was proposed by Vioux and co-workers [22,23]. The sol–gel process is considered non-hydrolytic when the organics are used instead of water as the oxygen-donor. Notably, NHSG process involves completely different gelation mechanisms, in which the structure, homogeneity, and surface properties of the resulting products can be manipulated by preparation conditions. 

Therefore, the porous WO_3_ films were prepared by an NHSG process and PEG was used as the structure-directing agent to form the porous structure. Considering that the structure and morphology of the WO_3_ films, which are correlated to the synthesis conditions, have a significant effect on their sensing performance. The effect of the aging time, PEG content, calcination temperature on structure, morphology, and NO_2_ sensing properties of WO_3_ films were investigated. The formation process of WO_3_ films prepared by NHSG method was also discussed.

## 2. Materials and Methods 

### 2.1. Materials

Tungsten hexachloride (WCl_6_, 99%), absolute ethanol (C_2_H_5_OH, 99.7%), dimethylformamide (DMF, C_3_H_7_NO, 99.7%), and polyethylene glycol (PEG, H(OCH_2_CH_2_)_n_OH, 99.5%, W = 1000) were purchased from Sinopharm Group, Shenyang, China. All reagents were analytic grade and used without further purification. The round glass substrates with a diameter of 2.5 cm were obtained from Kemiou Chemical Reagent Co., Ltd, Tianjin, China.

### 2.2. Synthesis of WO_3_ Porous Films

The WO_3_ porous films were prepared on glass substrates by a facile NHSG spin-coating method. First, the source used for the spin-coating process was prepared. In a typical procedure, the mixture solution of C_2_H_5_OH and DMF with the volume ratio of 1:2 was used as the solvent, 1 g WCl_6_ and a certain amount of PEG were completely dissolved in 4.5 mL of the mixture solution with the assistance of the ultrasonication. The as-prepared precursor solution was aged at 70 °C in a vacuum oven for hours to form the coating source. The glass substrates were cleaned with deionized water and ethanol several times, respectively, using an ultrasonication cleaner. Then, the cleaned substrate was set on the disk of the vacuum spin coater (VTC-100, Kejing Auto-instrument Co., Ltd, Shenyang, China). During the spin-coating process, 0.2 mL as-prepared coating source was dropped onto the substrate, which was rotated at 1000 rpm for 20 s in air at room temperature. After the spin-coating, the film was dried at 70 °C for 30 min in an electric heating oven. Then, the dried film was calcined at a pre-defined temperature for 5 h at a heating rate of 3 °C/min, and a layer of WO_3_ films was obtained from the glass substrate. The obtained samples were labeled as S(A, P, C), where A, P, and C represent aging time (h), PEG content (g), and calcination temperature (°C), respectively.

### 2.3. Characterizations

The crystallographic structure of WO_3_ films was determined by a PANalytical X’Pert Pro X-ray diffractometer, using a monochromatized Cu target radiation resource (λ = 1.5406 Å). The operating voltage and current were 40 kV and 40 mA, respectively. The surface morphology of the WO_3_ films was characterized using a ZEISS Ultra Plus field emission scanning electron microscope at an operating voltage of 20 kV. Transmission electron microscopy images of WO_3_ films were observed by a JEM2100 transmission electron microscope at an operating voltage of 200 kV. 

### 2.4. Fabrication and Measurement of the Gas Sensor

**Figure 1:**
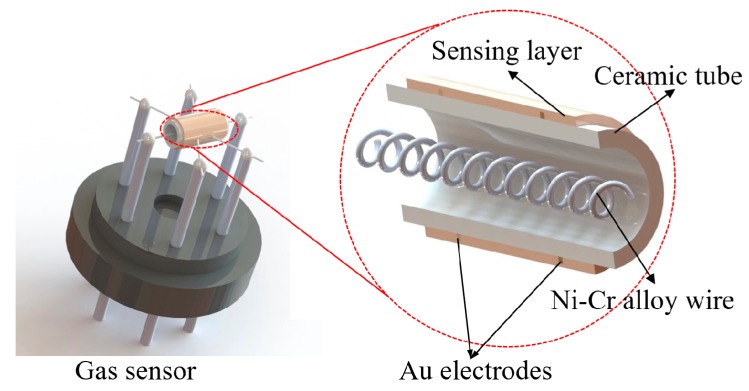
Schematic diagram of gas sensor device.

The gas sensor was fabricated as follows: The as-prepared WO_3_ films were dispersed in ethanol to form a homogenous paste, and then the resulting paste was coated on a cleaned alumina tube (4 mm in length, 0.8 mm in inner diameter, 1.2 mm in outer diameter) with a pair of Au electrodes and four Pt wires printed on its ends in advance. A spiral Ni−Cr alloy wire was inserted into the tube to control the operating temperature of the sensor. The structure of the sensor is schematically illustrated in Figure 1. Before gas sensing measurements, the sensor was aged at 300 °C for 24 h to improve the stability. The gas sensing performance of the sensor was investigated by a computer controlled static gas sensing test system (WS−30A, Winsen Electronics Technology Co., Ltd, Zhengzhou, China) as reported previously [24]. During testing, the initial resistance signal of the sensor in air (*R*_air_) was recorded first. Then, NO_2_ with a pre-defined volume was injected into the test chamber using a syringe. When the resistance of the sensor (*R*_gas_) reached a constant value, the chamber was opened to remove the NO_2_ to achieve the air−NO_2_−air cycles. The resistance of the sensor was recorded at 1 s intervals in the whole process. The response of the sensor was defined as *R*_gas_/*R*_air_. The response and recovery times of the sensor were defined as the times to reach 90% of the final equilibrium value.

## 3. Results and Discussion

### 3.1. Effect of Aging Time

**Figure 2:**
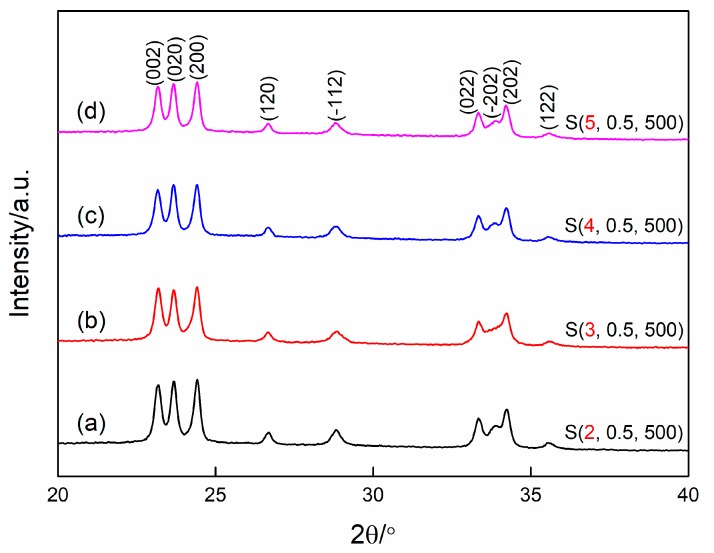
X-ray diffraction patterns of WO_3_ films prepared at different aging times. (**a**) 2 h, (**b**) 3 h, (**c**) 4 h, (**d**) 5 h.

It is well known that the properties of the sol used for the spin-coating process play a vital role in the quality of the films. Figure 2 shows the XRD patterns of WO_3_ films synthesized by using the sol with different aging times. It can be found that all the diffraction peaks can be indexed to monoclinic structured WO_3_ according to the standard JCPDS card no. 72–1465. The strong and narrow diffraction peaks indicate the excellent crystallinity of the samples. Further, slight changes of the diffraction peak of (−202) and the intensity ratio of the three main peaks located at 20–25° can be observed, indicating that the aging time of the precursor has an effect on the preferred growth orientation of the WO_3_ films. Additionally, no characteristic peaks from other impurity phases are detected in the XRD patterns, indicating all the samples are of high purity.

**Figure 3:**
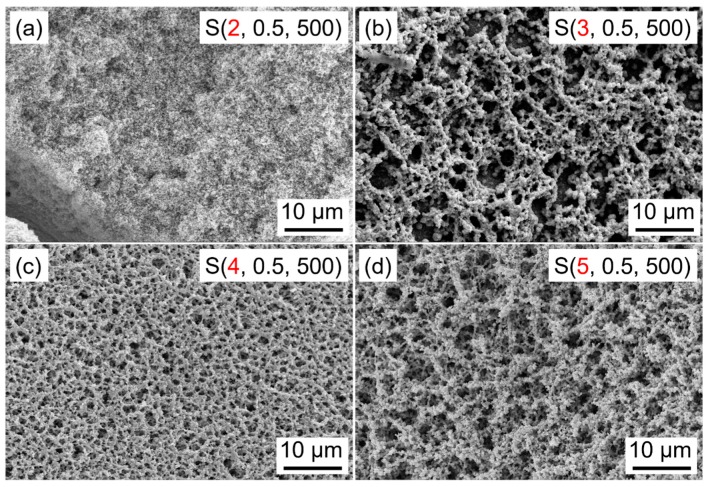
Scanning electron microscopy images of WO_3_ films prepared at different aging times. (**a**) 2 h, (**b**) 3 h, (**c**) 4 h, (**d**) 5 h.

The viscosity of the coating source, which is highly related to the aging time, has a great influence on the morphology and structure of the final obtained films. Figure 3 presents the SEM images of WO_3_ films synthesized by using the sol with different aging times. It can be clearly found that the porous films can be obtained in a wide range of aging times above 3 h. However, different porous structures and pore sizes are formed with different aging times. When the aging time of the precursor solution used for spin-coating is 2 h, WO_3_ film shows a relatively dense structure and a coarse surface morphology. As the aging time increases to 3 h, the network structured WO_3_ film with a high porosity is formed. The pores are irregular in shape with diameters ranging from several nanometers to micrometers. Furthermore, the pores are deep enough to form the channels through the entire films. By increasing the aging time to 4–5 h, porous structures are also observed on the entire surface of WO_3_ films. However, the size of the pores is significantly decreased, which may be mainly caused by the increased viscosity and the poor fluidity of the coating source from increasing the aging time.

**Figure 4:**
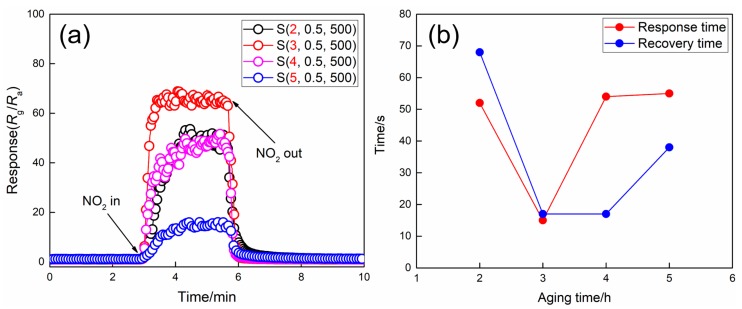
(**a**) Dynamic response/recovery curves and (**b**) response/recovery times of WO_3_ films prepared at different aging times upon exposure to 5 ppm NO_2_ at 100 °C.

Figure 4 shows the response/recovery curves and the response/recovery times of WO_3_ films prepared at different aging times upon exposure to 5 ppm NO_2_ at the operating temperature of 100 °C. Obviously, the sample S(3, 0.5, 500), which synthesized by using the coating source with the aging time of 3 h, has the highest response and the shortest response/recovery times compared with the other samples. In general, as for the gas sensor based on metal-oxide semiconductor materials, the higher specific surface area usually means the more active sites available for the adsorption and reaction of the gas molecules, leading to an enhanced sensor response. As for the porous film with a certain thickness, it is evident that the specific surface area increases as the pore size decreases. Based on SEM analysis, it can be concluded that the samples S(4, 0.5, 500) and S(5, 0.5, 500) with smaller pores should have larger specific surface areas than sample S(3, 0.5, 500). However, from the results of the gas sensing measurements shown in Figure 4a, samples S(4, 0.5, 500) and S(5, 0.5, 500) show significantly lower responses than the sample S(3, 0.5, 500) under the same condition. Therefore, the sensing efficiency of the porous films cannot be predicted by comparing their specific surface area only. On the one hand, the smaller the pores are, the larger the specific surface will be, namely, the more gas molecules can be adsorbed on the films. On the other hand, the size of the pores plays a key role in the penetration and diffusion of the gas molecules; the benefits of a high specific surface area should not be restricted by limitations in the accessibility of the film surface [25,26]. Thus, these two complementary aspects should be taken into consideration and a balance point should be found for the optimum gas sensing performance. Correspondingly, the shorter response/recovery times of the sample S(3, 0.5, 500) may be ascribed to its large pore size and the abundant channels that penetrate through the entire film, which can facilitate the diffusion and penetration of the NO_2_ molecules, resulting in the high response and short response/recovery times. As a result, the aging time of 3 h is chosen for the following experiments.

### 3.2. Effect of PEG Content 

**Figure 5:**
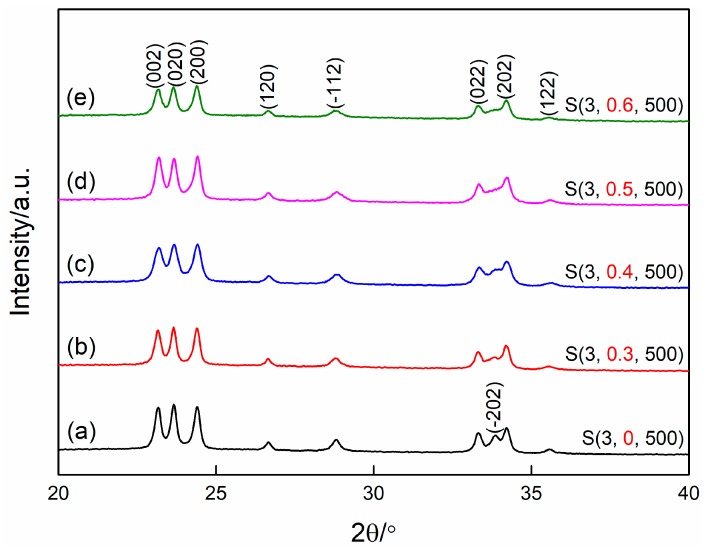
X-ray diffraction patterns of WO_3_ films prepared at different PEG contents (**a**) without PEG, (**b**) 0.3 g, (**c**) 0.4 g, (**d**) 0.5 g, (**e**) 0.6 g.

Figure 5 shows the XRD patterns of WO_3_ films synthesized at different PEG contents. It can be confirmed that all samples are well-crystallized with a monoclinic structure. Additionally, it is apparent that the diffraction peak of ($\bar{2}$02) disappears gradually when increasing the PEG content, demonstrating that the preferred growth orientation of the WO_3_ films is also correlated to the PEG content. Further, similar to Figure 2, the changes of the diffraction peaks located at 20–25° also confirm this.

**Figure 6:**
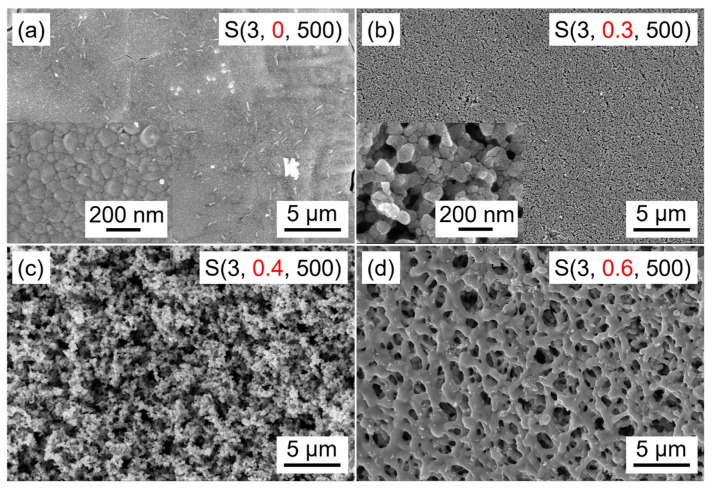
Scanning electron microscopy images of WO_3_ films prepared at different PEG contents. (**a**) without PEG, (**b**) 0.3 g, (**c**) 0.4 g, (**d**) 0.6 g.

PEG was used as a structure-directing agent for the formation of the porous structure of WO_3_ films. Figure 6 shows SEM images of WO_3_ films prepared at different PEG contents. As can be seen in Figure 6a, WO_3_ film prepared without PEG is compact and made up of spherical particles, which are 30–140 nm in diameter and contact closely with each other. In addition, some wrinkles and cracks on the surface of the film can be clearly observed. After 0.3 g PEG is added, the surface of WO_3_ film becomes smooth and some small-sized pores appear (Figure 6b). When the addition amount of PEG increases to 0.4 g, the film becomes loose and more pores are formed (Figure 6c). As the PEG content increases to 0.5 g, WO_3_ particles are cross-linked to each other and the porous network structured WO_3_ film is obtained (Figure 3b). At high PEG content of 0.6 g, as shown in Figure 6d, the film still retains the network structure with a relatively smooth surface, but no obvious particle boundaries are found on the skeleton of the network of WO_3_ film. Further, the porosity and the pore size obviously decrease compared to WO_3_ film prepared with 0.5 g PEG. It should be noted that the porosity of the films disappears, and the films become gradually more compact with the further increase of PEG content, although the corresponding results are not shown here. It may be explained that the combination strength of PEG is stronger than that of W oligomers and PEG; thus, the excess PEG will self-assemble and finally separate out from the sol [27]. In conclusion, the porous structure of WO_3_ film is closely related to PEG content, and it is suggested that better porous WO_3_ film can be obtained when the added PEG amount is 0.5 g under the given conditions.

**Figure 7:**
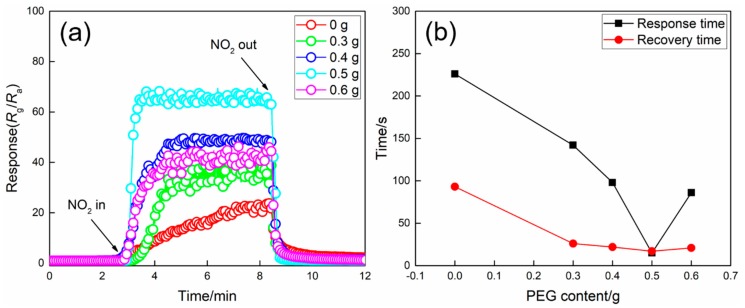
(**a**) Dynamic response/recovery curves and (**b**) response/recovery times of WO_3_ films prepared at different PEG contents upon exposure to 5 ppm NO_2_ at 100 °C.

The dynamic responses of WO_3_ films prepared at different PEG contents upon exposure to 5 ppm NO_2_ at 100 °C are shown in Figure 7a. It can be observed that the resistances of all the samples increase to reach a stable value when NO_2_ is introduced to the test chamber and recover to their initial states after NO_2_ is removed, revealing the good reproducibility of the samples. Furthermore, it is found that the response of WO_3_ film enhances gradually with the increase of PEG content from 0 to 0.5 g. In detail, the responses are 21, 37, 49 and 64 for WO_3_ films prepared with 0, 0.3, 0.4 and 0.5 g PEG, respectively. However, the response reduces with the further increase of PEG content. The results may be ascribed to the gradual increase in porosity and the formation of the network structure by increasing the PEG content. And the decrease of the response when PEG content increases to 0.6 g can be explained by the significantly decreased porosity and pore size. The corresponding response/recovery times are presented in Figure 7b. Similar to the relationship between the response and PEG content, the response/recovery times reduce when increasing PEG content if the PEG content is less than 0.5 g, and then increases with the further increase of PEG content. The results are consistent with the variation of the porosity and pore size of WO_3_ films prepared at different PEG contents.

### 3.3. Effect of Calcination Temperature

**Figure 8:**
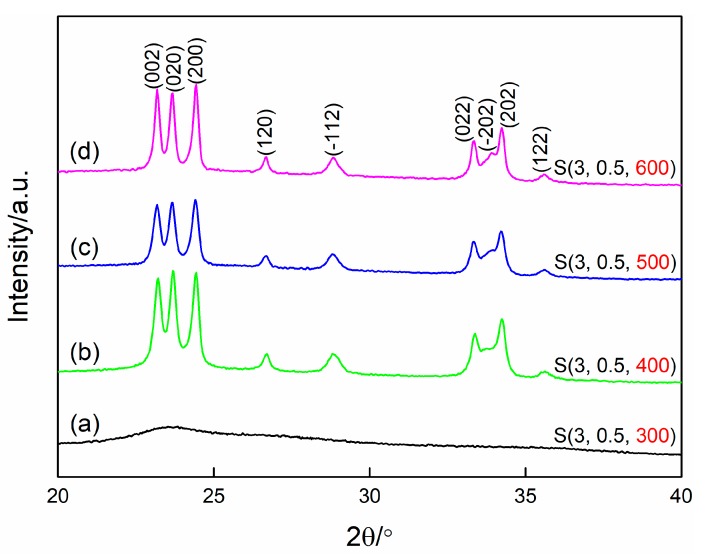
X-ray diffraction patterns of WO_3_ films prepared at different calcination temperatures. (**a**) 300 °C, (**b**) 400 °C, (**c**) 500 °C, (**d**) 600 °C.

As reported before, the calcination process has an important effect on the morphology, structure, and crystallinity of the final products when the sol–gel method is employed [28,29]. Figure 8 presents the XRD patterns of WO_3_ films prepared at different calcination temperatures. It can be seen in this figure that only a broad diffraction peak with low intensity is observed for the sample S(3, 0.5, 300), revealing that WO_3_ film prepared at the calcination of 300 °C is in an amorphous state. The result may be due to the fact that the organics cannot be removed completely at 300 °C. However, with the increase of the calcination temperature up to 400 °C, the sharp and strong peaks of monoclinic structured WO_3_ (JCPDS card no. 72–1465) can be clearly observed, indicating that the well-crystalline WO_3_ films are formed when the calcination temperature is above 400 °C. No other crystal phase is observed in the XRD patterns of Samples S(3, 0.5, 400), S(3, 0.5, 400), and S(3, 0.5, 400), demonstrating that the single-phase WO_3_ films can be obtained at the calcination temperature range of 400 to 600 °C. Additionally, although the samples S(3, 0.5, 400), S(3, 0.5, 400), and S(3, 0.5, 400) all show good crystallinity, it can be found that the peak of ($\bar{2}$02) increases while the peak of (020) decreases gradually when increasing the calcination temperature, which demonstrates that calcination temperature has a significant effect not only on the crystallinity but also on the preferential growth of the WO_3_ films.

**Figure 9:**
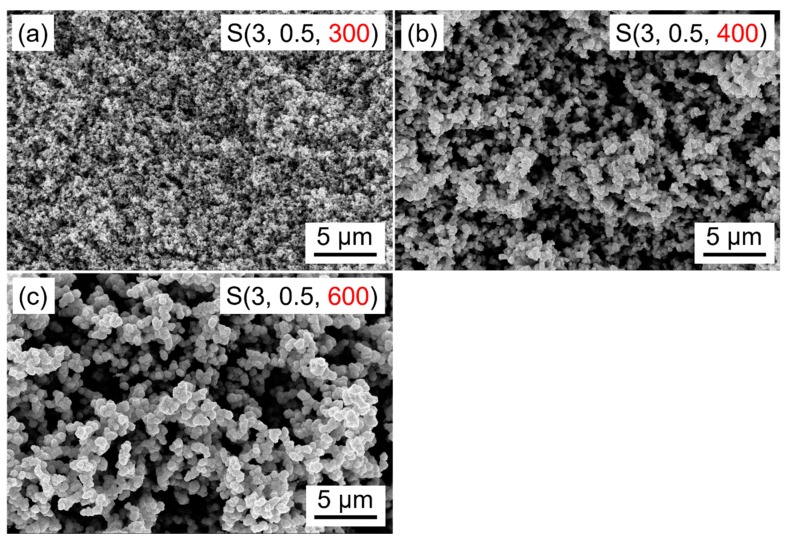
Scanning electron microscopy images of WO_3_ films prepared at different calcination temperatures. (**a**) 300 °C, (**b**) 400 °C, (**c**) 600°C.

The relationship of the calcination temperature and the morphology of WO_3_ films were also investigated by SEM, and the corresponding results are shown in Figure 9. With the removal of the organics and the transformation from the amorphous to monoclinic structure, the films become porous gradually. In addition, it is apparent that the particle size increases with the calcination temperature. This is expected since the higher temperature gives more energy for particles growth. 

**Figure 10:**
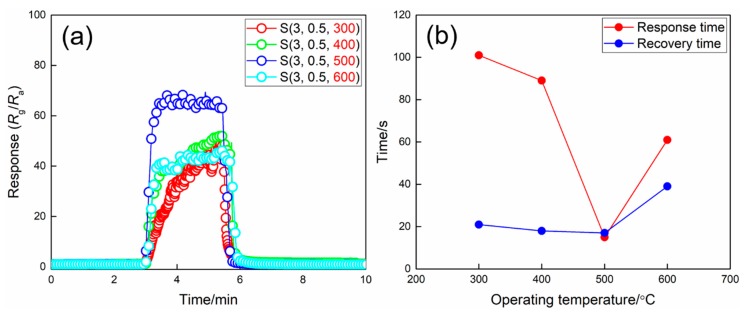
(**a**) Dynamic response/recovery curves and (**b**) response/recovery times of WO_3_ films prepared at different calcination temperatures upon exposure to 5 ppm NO_2_ at 100 °C.

The dynamic responses and the response/recovery times of WO_3_ films prepared at different calcination temperatures are shown in Figure 10. Based on the results of XRD measurement, the sample S(3, 0.5, 300) is amorphous; however, it can be found from Figure 10 that the film still has a high NO_2_ response of 45 to 5 ppm NO_2_ at 100 °C. The responses of sample S(3, 0.5, 400), S(3, 0.5, 500), and S(3, 0.5, 600) are 54, 64, and 45, respectively. The corresponding response/recovery times are 89/18, 15/17, 61/39 s, respectively. In theory, the smaller particles are always expected for a larger specific surface area and thus more active sites, leading to a higher sensor response. However, Yamazoe et al. found that the higher H_2_ and H_2_S responses could be obtained using the SnO_2_ films with the larger particle size while keeping the thickness of the films unchanged [30,31]. As we know, gas sensing is a very complicated process; thus, many parameters of the sensing film, such as porosity, particle size and interconnectivity, and pore size, should be taken into consideration. Michael pointed out that an increase in average particle size of the porous film will result in larger pores; thus, the diffusivity of the analyte gases will be enhanced, and the higher response can be obtained [25]. Meanwhile, the interconnectivity of the particles also has a great effect on the gas sensing performance of the porous film, because the overall electronic conductance requires sufficient contact between the neighboring particles in order to facilitate percolation paths through the entire sensing layer [32]. As can be seen in Figure 9, it is obvious that the particle size as well as the particles’ interconnectivity increase when increasing the calcination temperature, which is also beneficial to the gas sensing process. However, on the other hand, the particle size is negatively correlated to the specific surface of the film, namely, the increase of the particle size will significantly decrease the specific surface area, which is disadvantageous for gas sensing. Therefore, as shown in Figure 10, the response and response/recovery times increase when increasing the calcination temperature, and then decrease by further increase of the calcination temperature. The results are closely related to the different structure parameters of the films that were prepared at different calcination temperatures. It can be found that the film calcined at 500 °C is more proper for the application of NO_2_ sensing.

In summary, the aging time of the precursor solution, PEG content, and calcination temperature play a vital role in the structure, morphology, and crystallinity of WO_3_ films. Although these three parameters are interdepended, according to the experimental results, it can be concluded that: (1) The aging time has a more significant effect on the pore size of the film; (2) PEG, as a structure-directing reagent, plays an important role in the formation of the porous structure; (3) calcination temperature mainly affects the particle size and the crystallinity of the film. As for the gas sensing application, because many influence factors are highly interdependent, a compromise among these considerations may be necessary during the film synthesis process. In this study, the sample S(3, 0.5, 500) is a better choice for NO_2_ detection.

**Figure 11:**
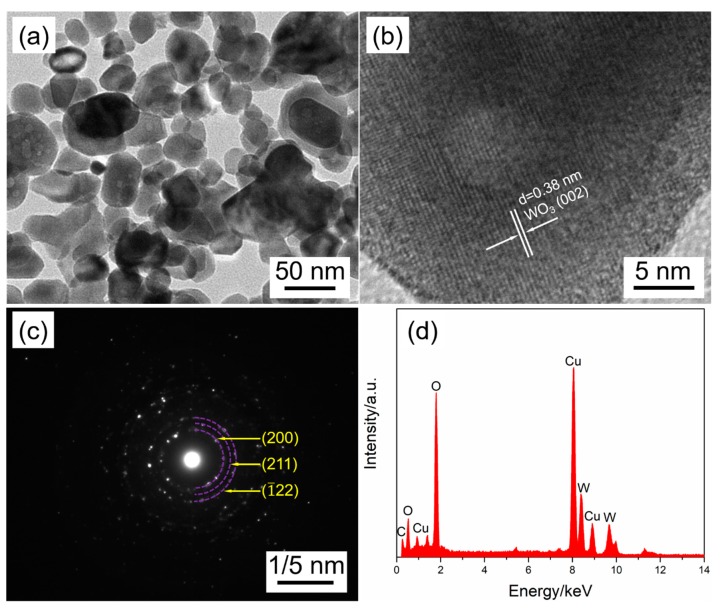
(**a**) Transmission electron microscopy, (**b**) HRTEM, and (**c**) SAED images of the sample S(3, 0.5, 500). (**d**) The corresponding EDX spectrum.

Finally, further detailed morphology and structure characteristics of the sample S(3, 0.5, 300) were investigated using TEM measurement. It can be seen from Figure 11a that the as-prepared WO_3_ porous films are made up of WO_3_ nanoparticles with the diameter of 20~80 nm, which is in agreement with the SEM results. The HRTEM images displayed in Figure 11b indicates that the *d*-spacing value of the lattice fringe is 0.38 nm, which corresponds to (002) lattice spacing of monoclinic structured WO_3_. The corresponding SAED pattern (Figure 11c) exhibits diffraction rings, revealing the polycrystalline structure of WO_3_ film. The EDX spectrum is presented in Figure 11d, except for the C and Cu signal peaks arising from the carbon coated copper mesh used for supporting the samples during the measurement; the other peaks are assigned to W and O element, which further confirms the high purity of the obtained WO_3_ films.

**Figure 12:**
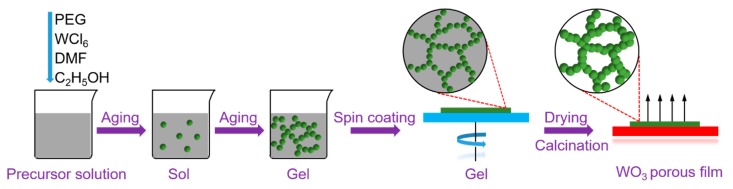
Schematic diagram of the formation process of WO_3_ porous film.

The formation process of the porous WO_3_ films is explained as follows and illustrated in Figure 12. Ethanol was used instead of water as the oxygen donor in the experiment. Based on a previous report [33,34], the nucleophilic substitution reactions occur with the elimination of HCl according to Equation (1), when the WCl_5_ and PEG are dissolved in the mixture solution of DMF and C_2_H_5_OH. At the same time, the condensation of the monomers takes place to form the particles and chains according to Equation (1,2) during the aging process at 70 °C. When increasing the aging time, condensation reactions continue. Meanwhile, the particles and chains grow followed by the cross-link with each other, resulting in the formation of the network structure and the transformation from sol to gel gradually. During this process, PEG plays the role of structure-directing reagent in the formed inorganic–organic network structure through the linked actions. In the subsequent spin-coating, drying, and calcination process, the residual solvents and organics are removed from the materials and the porous WO_3_ films are obtained.

WCl_6_ + xC_2_H_5_OH → WCl_6-x_(OC_2_H_5_)_x_ + xHCl↑ (1)

2WCl_6-x_(OC_2_H_5_)_x_ → (OC_2_H_5_)_x-1_Cl_6-x_W–O–WCl_5-x_ (OC_2_H_5_)_x_ + C_2_H_5_Cl↑ (2)

WCl_n−x_(OC_2_H_5_)_x_ → (OC_2_H_5_)_x−1_Cl_n−x_W–O–WCl_n−1−x_(OC_2_H_5_)_x_ → ······ →–W–O–W–Cl–W–(3)

## 4. Conclusions

Tungsten trioxide films with different porosity and microstructure were synthesized on glass substrates by a NHSG spinning-coating process, in which two major reactions including nucleophilic substitution and condensation were involved. The aging time of the precursor solution, PEG content, and calcination temperature had a significant effect on the microstructure of WO_3_ films. The aging time, PEG content, and calcination temperature played a more important role in pore size, formation of the porous structure, particle size, and crystallinity of the films, respectively. The gas sensing measurements indicated that the NO_2_ sensing properties of WO_3_ films are strongly correlated to their microstructure. Further, WO_3_ films that synthesized at the aging time of 3 h, PEG content of 0.5 g, and calcination temperature of 500 °C exhibited the highest NO_2_ response and shortest response/recovery times. Under this synthesis condition, the prepared WO_3_ films had a network structure with high porosity.
